# Ebola and Its Global Research Architecture—Need for an Improvement

**DOI:** 10.1371/journal.pntd.0004083

**Published:** 2015-09-25

**Authors:** David Quarcoo, Dörthe Brüggmann, Doris Klingelhöfer, David A. Groneberg

**Affiliations:** 1 Department of Preventive Medicine, Institute of Occupational Medicine, Social Medicine and Environmental Medicine, Goethe-University, Frankfurt, Germany; 2 Department of Obstetrics and Gynecology, Keck School of Medicine, University of Southern California, Los Angeles, California, United States of America; Armed Forces Health Surveillance Center, UNITED STATES

## Abstract

The current Ebola outbreak poses a threat to individual and global public health. Although the disease has been of interest to the scientific community since 1976, an effective vaccination approach is still lacking. This fact questions past global public health strategies, which have not foreseen the possible impact of this infectious disease. To quantify the global research activity in this field, a scientometric investigation was conducted. We analyzed the research output of countries, individual institutions and their collaborative networks. The resulting research architecture indicated that American and European countries played a leading role regarding output activity, citations and multi- and bilateral cooperations. When related to population numbers, African countries, which usually do not dominate the global research in other medical fields, were among the most prolific nations. We conclude that the field of Ebola research is constantly progressing, and the research landscape is influenced by economical and infrastructural factors as well as historical relations between countries and outbreak events.

## Author Summary

For the first time in the history of the disease, the Ebola virus left its local setting and affected people not only in isolated rural areas, but reached larger towns and cities leading to worldwide repercussions. This development prompted a joint global response to this health threat. This encompassed not only immediate relief efforts, but also an up search in global research work. In this study, the scientific output in Ebola research available in one of the mayor medical search platforms was characterized. We studied among others the origin of research, the collaboration between countries and the research topics. Partly, the obtained data was weighted against economic parameters. We attained a detailed map of the research activities from the discovery of Ebola in 1976 up to today. Our research provides the first overview of the worldwide Ebola research output. It might help stakeholders in Ebola research to better plan investigations with a global perspective.

## Introduction

No other infectious disease event has captured the attention of the international health community in recent years like the Ebola outbreak. The current epidemic started in December 2013 leading to over 26.000 infected patients and more than 10.000 deaths [1] The outbreak reached global dimension as hospitals in the United States of America (US) and Europe are now treating patients returning from health missions in Ebola affected countries [2].

28 outbreaks were documented since 1976, which all, except the recent one, occurred in isolated regions. During the first epidemic in the Democratic Republic of Congo and the Sudan the Ebola virus was identified as a non-segmented, negative-strand RNA virus and placed within the *Filoviridae* family. Since then five distinct virus species were distinguished. Although the pathogenesis of the Ebola virus is intensively investigated worldwide, the undisputed identification of the natural reservoir has not been successful yet. Bats were implicated as a possible host for the Ebola as well as the related Marburg virus [3].

Despite the short time span since the discovery of the Ebola virus, scientists have authored a substantial body of related scientific literature worldwide. Nevertheless, it should be an ethical obligation of all industrialized countries to invest future capacities in research of this life-threatening disease and vaccination strategies as one of the most effective means to fight infectious diseases [4,5].

In order to cast a first light on the question of global research activity in this field since 1976, we present a combined scientometric and density equalizing study. It encompasses scientometric tools and advanced visualizing techniques such as density equalizing mapping [6] and draws a sketch of the global Ebola research architecture over the past 40 years. Scientometric analysis of the scientific output of individuals, institutions and countries is represented in the number of publications as well as citations and their bibliometric parameters. Density equalizing map projections (DEMP) are used as a state of the art technique to demonstrate the global architecture on the research output via distorted maps.

## Methods

The data was analyzed using scientometric methods developed in the NewQIS project as previously described [7]. The analyzed data was retrieved from the Thomson Reuters Web of Science database (WoS) using the search term “Ebola” in the Science Citation Index Expanded (SCIE) and the Social Sciences Citation Index (SSCI) (time frame between the first description of the virus in 1976 and 2014). To limit our search to the original research articles, we used the WoS´s option for selecting the document type and included only “articles” in the analysis. Data was processed as previously described using a combination of scientometric tools with density equalizing mapping [8]. For the generation of density equalizing mapping, the Gastner and Newman’s algorithm was employed [6]. As parameters, citation quantities were determined using the “citation report” function (number of citations per article, the citation rate of countries, and authors), H-indices [9] along with other general operating figures (year of publication, country of publication, co-operations between different countries, language of publication, document types, subject areas, and journals). Also, author analysis, subheading-terms, and individual subject areas were examined. To evaluate the quality of a country’s publication output, we assessed the citation rate and the modified H-index [9].

## Results

### Global research activity

The total numbers of publications in the database added up to 2482 (search term “Ebola” only as title word) and 3081 (search term “Ebola” also in keywords and abstract) starting from 1976 with a steady yearly increase in publication activity until 2014. Ebola research originated from 78 countries. Research groups based in the USA published most research with 1367 articles (44,4% of all determined articles), followed by groups from Germany (272 articles, 8.8%), Canada (202 articles, 6.6%), France (179 articles, 5.8%), United Kingdom (167 articles, 5.4%), Japan (157 articles, 5.1%), Russia (84 articles, 2.7%), Gabon (70 articles, 2.3%), Belgium (58 articles, 1.9%) and Switzerland (51 articles, 1.7%). Each percent value stands for the part of the overall Ebola research output that was retrieved via the WoS.

The rest of articles originated from the remaining 68 countries that are involved in Ebola research.

**Fig 1:**
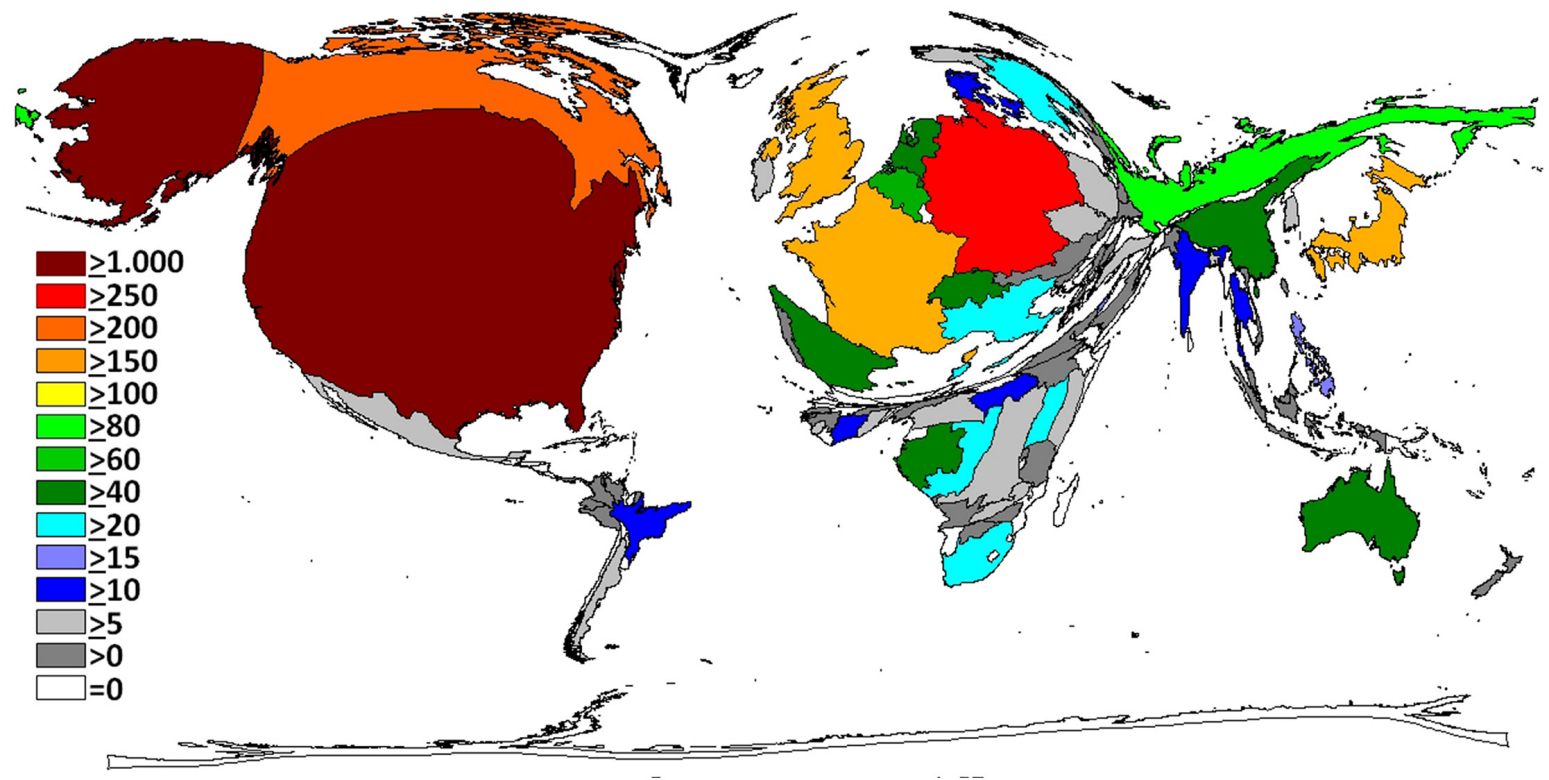
Density equalizing maps of the total number of Ebola related publications.

**Fig 2:**
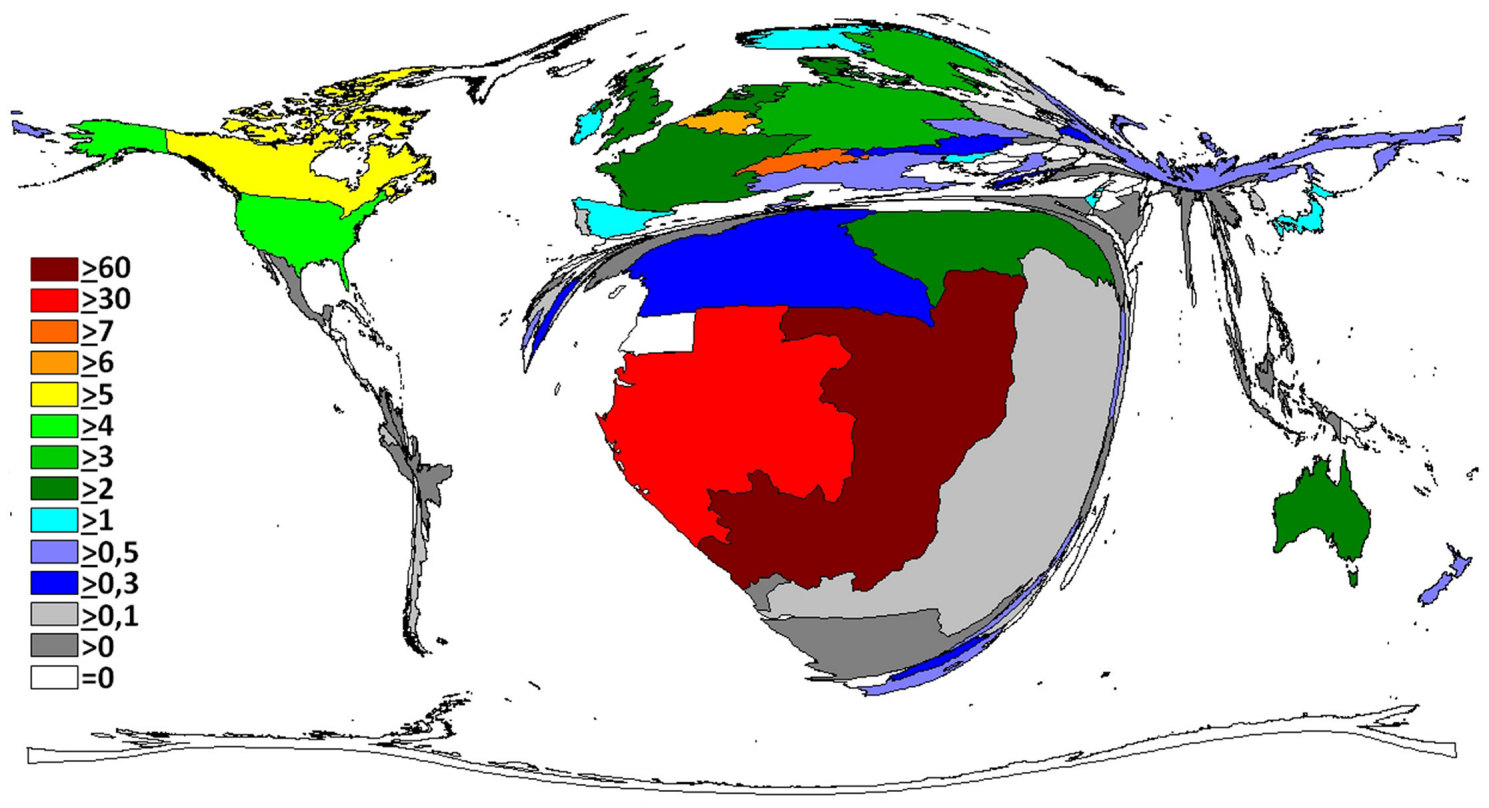
Density equalizing map of the Ratio of the number of publications to the number of inhabitants.

**Fig 3:**
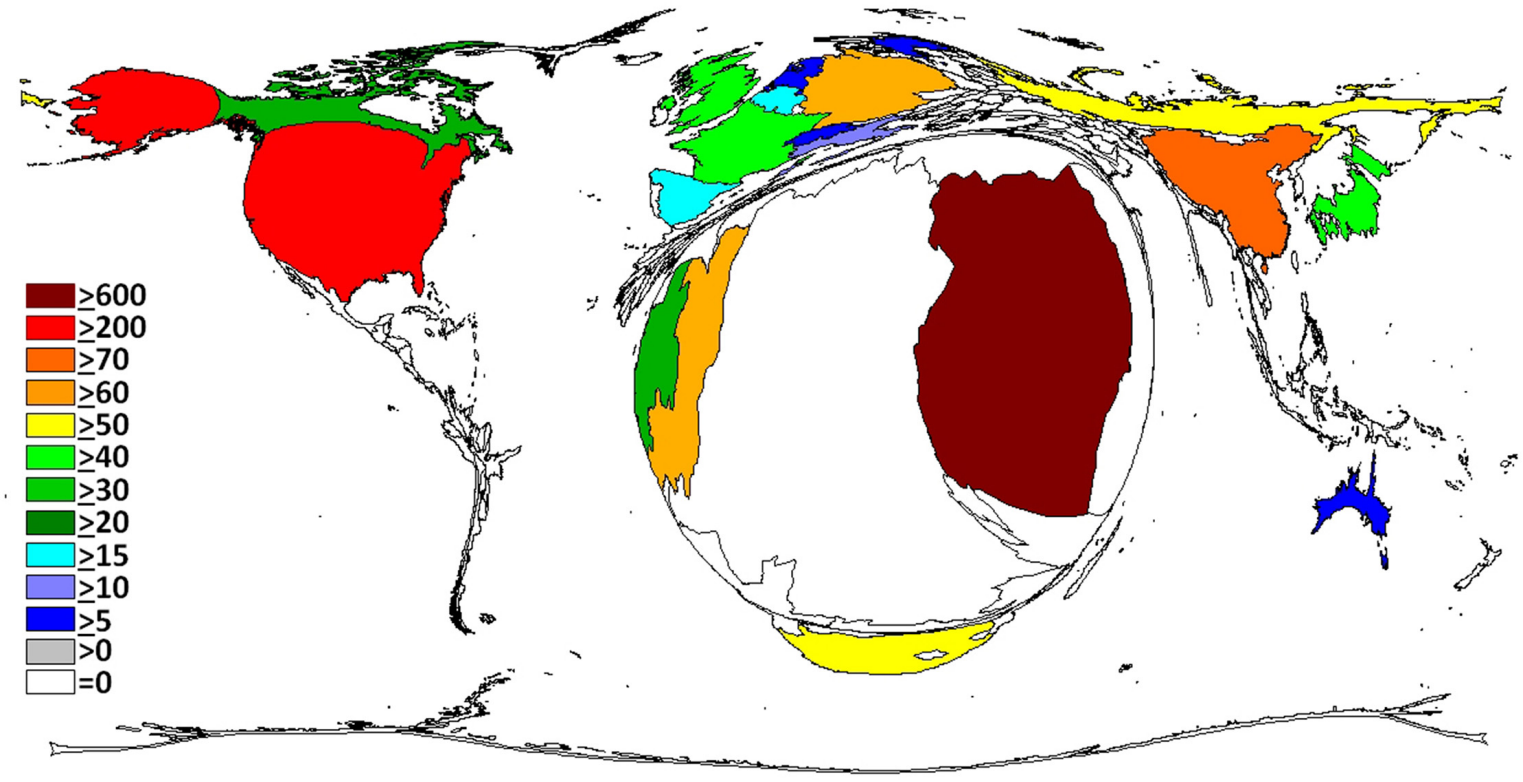
Density equalizing map of the Ratio of the number of publications and the Gross Domestic Product (GDP) per Capita x 10,000. Threshold: 30 publications.

Overall, North American and European countries took a lead position. The density equalizing mapping of the world shows that research activity translated into a distorted global architecture (Fig 1). African countries affected by Ebola cases exhibited a relatively low activity but were present. The continents South America, and Asia almost disappeared from the cartogram. When relating these operating figures to population numbers (Fig 2), we found that—besides the most active nations (US, European)—smaller African countries such as Gabon, Republic of Congo, Central African Republic and Cameroon gained importance. Uganda, Republic of Congo, Gabon and South Africa reached increased ratios regarding their research activity adjusted to Gross Domestic Product (GDP, Fig 3). We did not find any association between the death rate and the research output on a regional scale.

### Citation parameters

**Fig 4:**
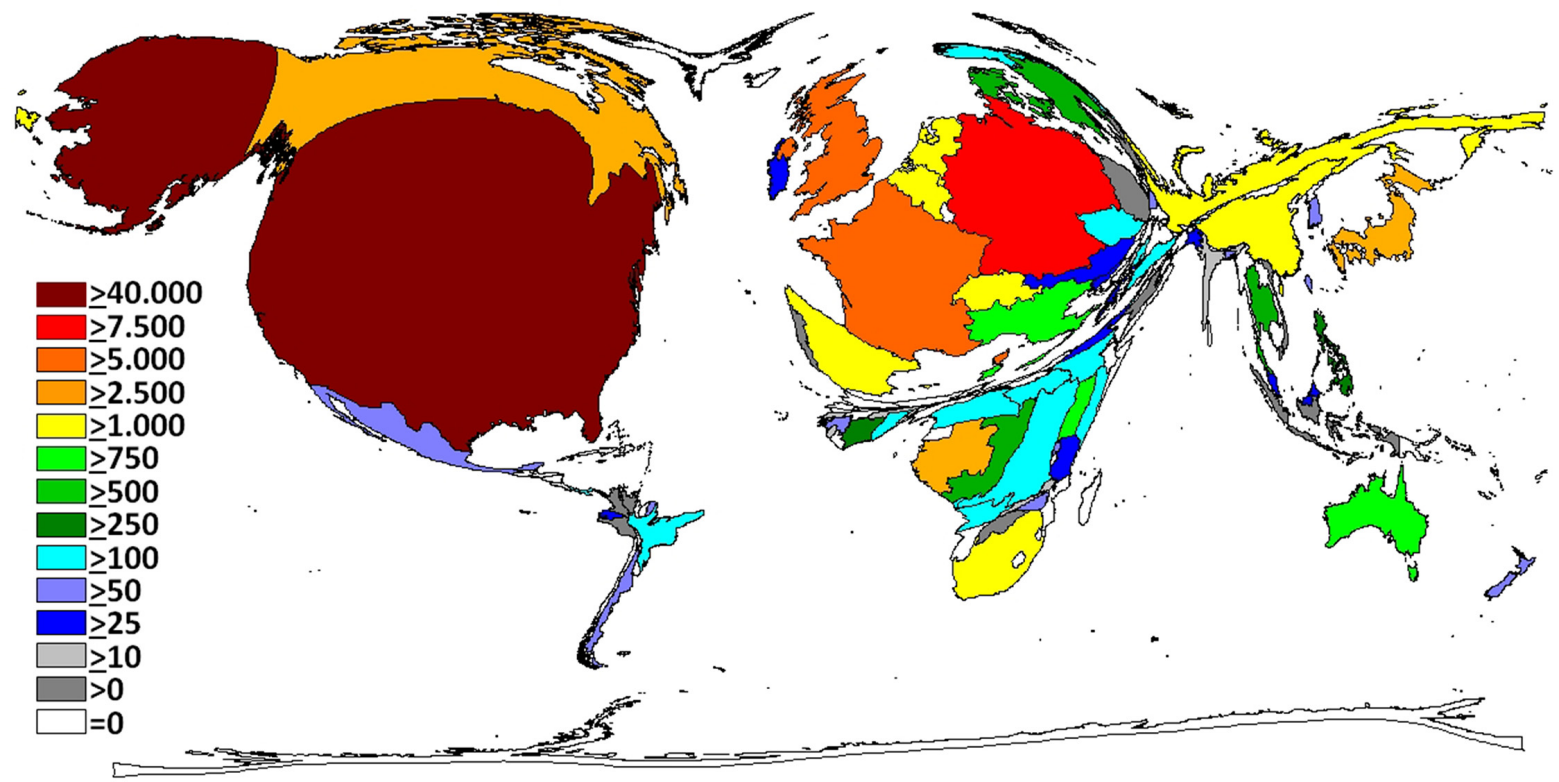
Density equalizing map of the total number of citations.

**Fig 5:**
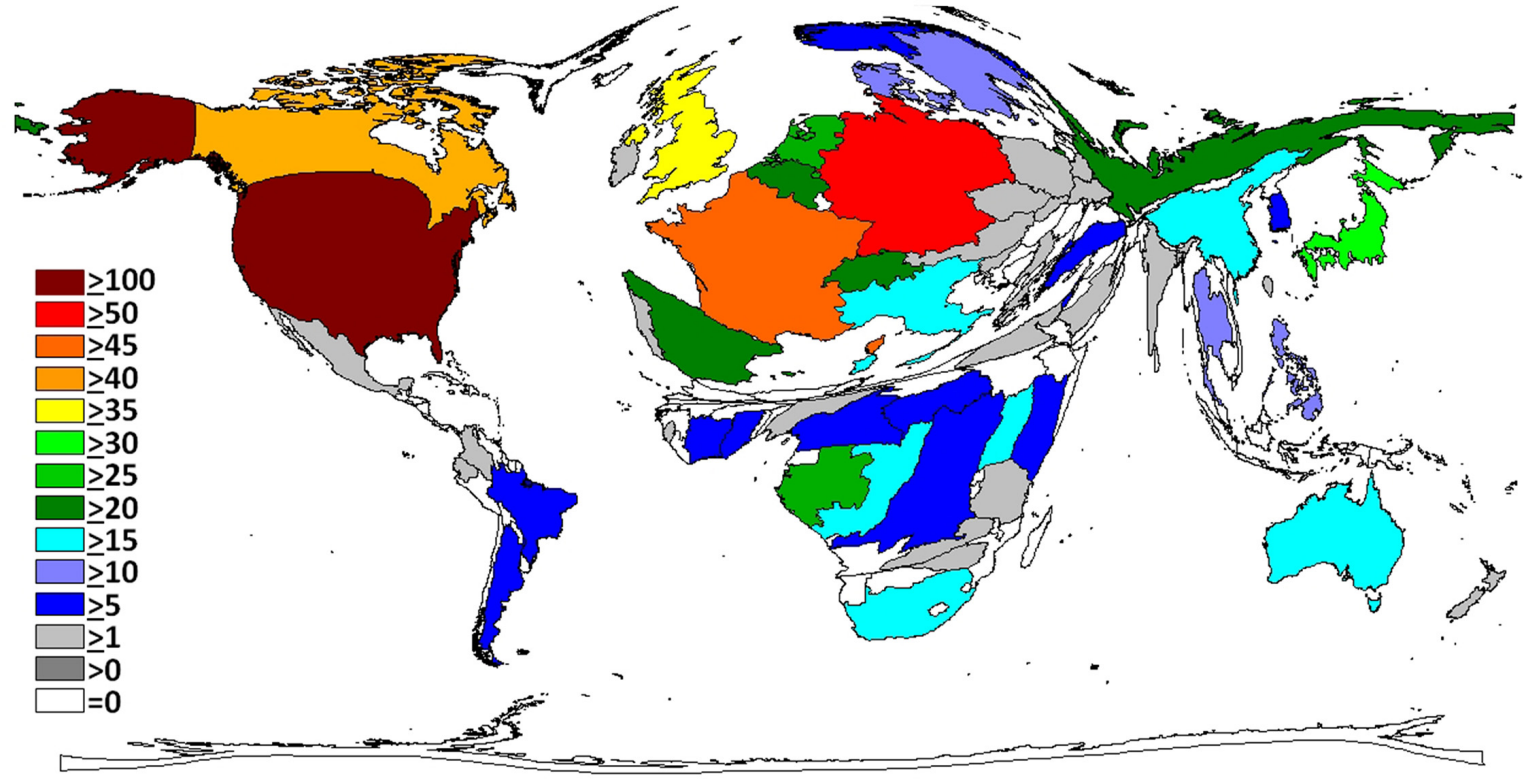
Density equalizing map of the modified h-index.

With reference to the citation rate (CR = average ratio of citations per publications), Gabon was ranked first (CR 43.3) followed by western countries (Switzerland = CR 34.4; Germany = CR 33.2; France = CR 32.9; US = CR 32.6; and UK = CR 30.1) (Fig 4). Focusing on the modified h-index (country specific and thematically related), the US exhibited the highest h-index (102) followed by Germany (55), France (46), Canada (41), UK (37), Japan (32), Gabon (26), Switzerland (24), Belgium (23) and Russia (20) (Fig 5).

### Global cooperation

**Fig 6:**
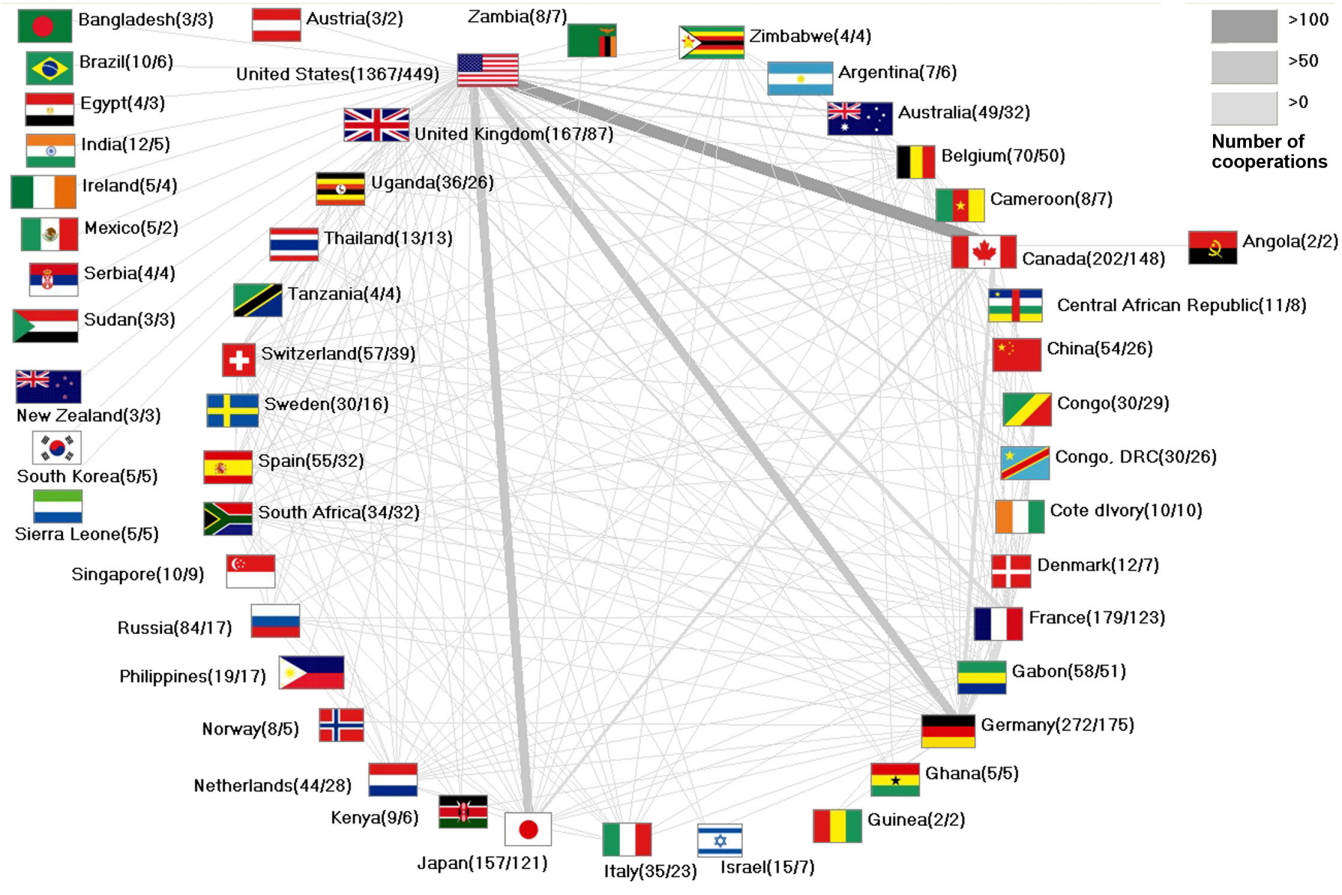
International cooperations. Threshold: 2 cooperation articles. Numbers in brackets (number of publications / number of collaboration articles).

Ebola research has been characterized by strong international cooperations since its beginnings. Although most global research involved cooperation with US-American research groups (449 collaborative articles), the overall rate of US collaborative publications in relation to the total US output activity with 1367 articles in total (33%) was relatively low compared to other countries (Fig 6).

Most frequent research partners of the USA were Canada (121 US-Canadian collaborative articles), Japan (99 US-Japan collaborative articles) and Germany (88 US-German collaborative articles). Canada also played an important role with 148 collaborative articles altogether conducted with a total of 11 countries (73% of all Canadian articles), followed by Japan with 121 collaborative articles in total (77% of all Japanese articles), Germany with a total of 175 collaborative articles (64%), France with 123 collaborative article all in all (69% from all French articles), the UK with 87 collaborative articles in total (52% of all UK articles) and Belgium with 50 collaborative articles (71% of all Belgian articles).

Due to the fact that most Ebola outbreaks were geographically defined to the African continent we found particularly strong cooperations of France with Gabon with 38 collaborative articles (USA and Gabon have 19 common articles; Germany and Gabon have 14 common articles). Gabon has worked out 87% of the overall publication output together with other countries. The collaboration between the USA and Uganda followed with 21 collaborative articles. Uganda has written 26 together with other countries (72% of all Ugandan articles). South Africa published 94% of the overall research output as collaborative articles and Congo nearly 97%.

**Fig 7:**
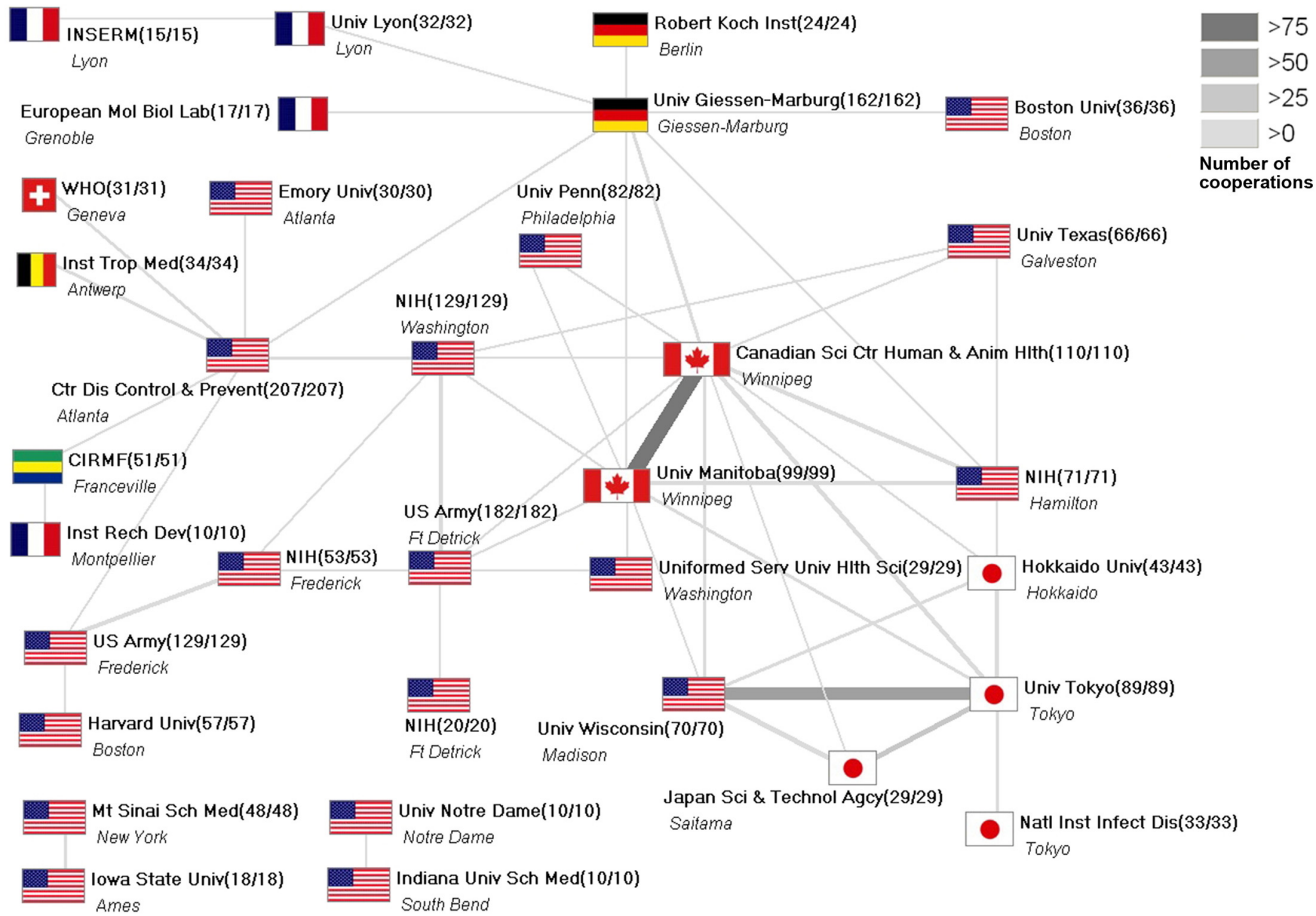
Institutional research networks in the field of Ebola research. Threshold: 10 cooperation articles. Numbers in brackets (number of publications / number of collaboration articles).

On an institutional level most cooperate publications were produced between the Canadian Science Center of Human and Animal Health and the University of Manitoba. Both located in Winnipeg and have an overlap of staff working in both facilities. Also, numerous cooperations were found between research institutions within the US. Laboratories with the highest biosafety level clearance were available in 13 of the leading 33 cooperating institutions (Fig 7).

### Categories of research

**Fig 8:**
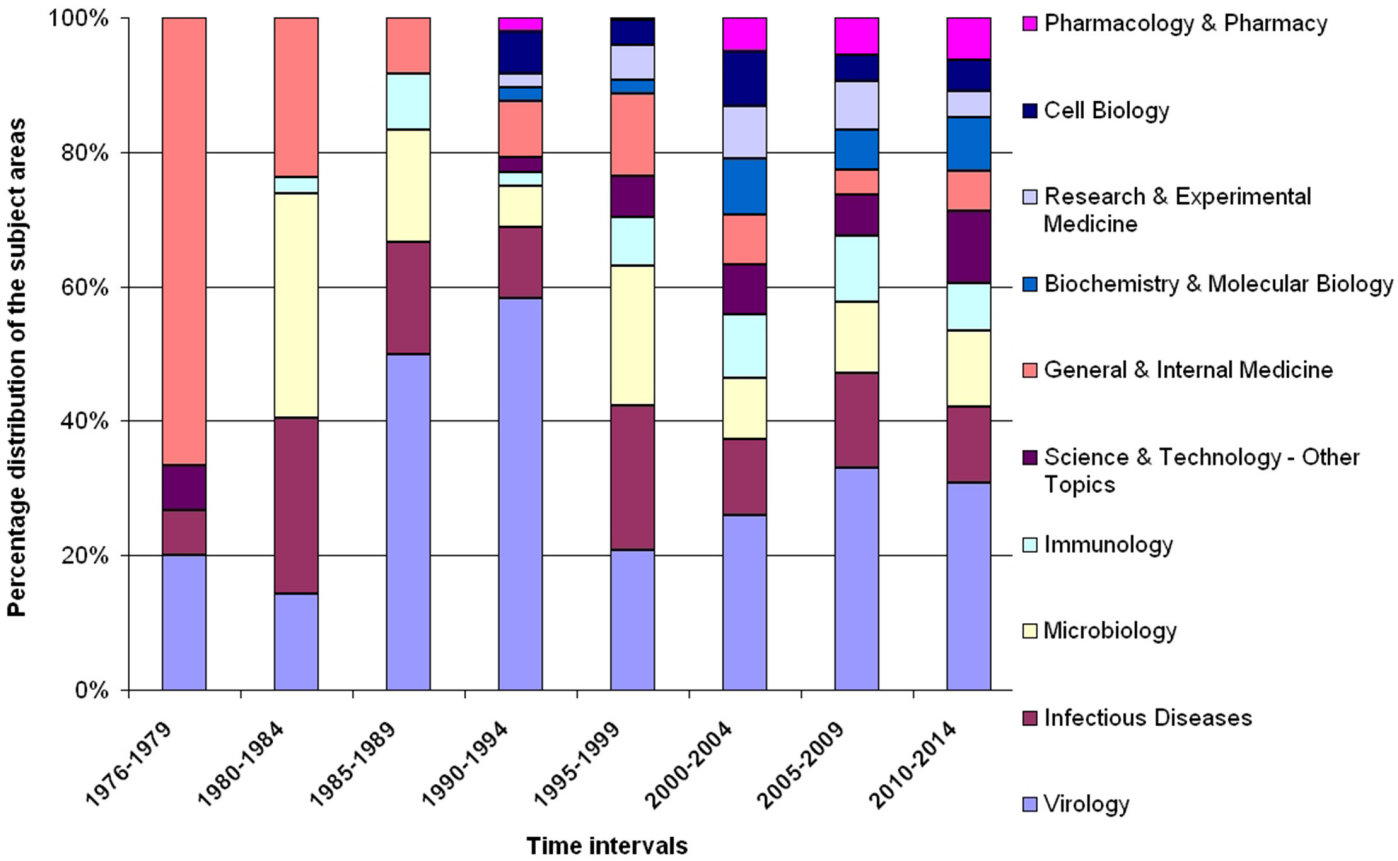
Relative proportional distribution of the most assigned subject categories over the time. 4 year intervals beginning in 1976.

Subject categories are defined classes of themes indicating a general area of science. For the field of Ebola research, they were determined and depicted in four year intervals (Fig 8). The research interest shifted from a predominant focus on general and internal medicine to a much more diversified picture covering subject categories in immunology, cell biology, pharmacology, experimental medicine, biochemistry and molecular medicine. We did not find a remarkable attribution to the category of public health.

## Discussion

In order to give an overview over the global Ebola research architecture, the current study employed the Web of Science and conducted the first analysis of the research output for the entire field since 1976. Other than the majority of infectious diseases that have been discussed in the scientific literature for more than a century (e.g. yellow fever or dengue [10,11]) the advent of Ebola research is recent and started from its first description in 1976 [12]. Therefore, Ebola research offers a rare opportunity to observe the entire development of a scientific field since all related publications are achieved in electronically accessible scientific databases. Overall, we found that the number of publications was constantly low in the years after 1976 and remained on a level of up to 20 publications per year. This is surprisingly low for a dangerous infectious disease but understandable since a biosafety level 4 laboratory is needed to carry out the research. Coinciding with the reemergence of the virus in Kikwit (located in the Democratic Republic of Congo) [13], research activity was evidently enforce dafter 1995.

With regard to the landscape of Ebola research analyzed in this study, US-American institutions contributed the largest amount of international research. This finding again demonstrates the leading role of the USA in science, as it is present in almost all other areas of biomedical research [14]. We also showed a particular strong involvement of German institutions in Ebola research. This might be explained by the local availability of numerous suitable research facilities that were established after the first European outbreak of the Marburg virus. This virus is termed the “forgotten cousin” of Ebola and also causes life threatening hemorrhagic fever [15]. The application of the number of the total population and socio-economic parameters changed the ranking of the nations: Whereas the apparent superiority of the USA research was put in perspective, the African countries that are traditionally most affected by the disease such as Gabon, Republic of Congo, Central African Republic, Cameroon and Uganda increased their ranking.

Only a few studies evaluated the worldwide research efforts in the field of tropical diseases. These also found a predominance of North American and European countries regarding overall research activity. As examples of countries where particular diseases play a significant role for the health of the public, only Brazil, where yellow fever is endemic [16], as well as Brazil and India, which are affected by Leishmaniasis, were among the 10 most prolific countries [17]. Even high prevalent infections such as malaria have not lead to a increased global impact of research originating from African countries [18].

From its first appearance in scientific databases Ebola research was characterized by a high percentage of collaborative studies as demonstrated in our study. It has been shown numerously that involved scientists benefits from the international cooperation [19]. Although the US is the favorite global cooperation partner for scientists from other countries, it had the lowest cooperation ratio in regard to its own overall publication activity. This might be caused by the fact that American scientists cooperate to a large extend with national colleagues due to the efficient and well-funded academic structure that is present in the US.

When focusing on the collaboration of institutions committed to Ebola research, we found a network that favors only a limited amount of institutions. This might be explained by the necessary prerequisite of Ebola research. The risks involved in handling the virus require the maximum biosafety level [20]. Research facilities that provide these resources are sparsely distributed throughout the world and its highest numbers are found in the US–the leading country of Ebola research output.

Subject categories in health research represent the interest of scientist in different aspects of a disease. In the beginning of Ebola research, publications dominated that were attributed to the categories of microbiology, internal medicine and virology. Then the field became more diverse including other subjects such as immunology, cell biology, pharmacology, and biochemistry. Research in the field of public health encompasses society-based measures to combat diseases. We want to point out that a lack of publication output regarding public health topics is apparent in the field of Ebola research, which is in sharp contrast to other tropical diseases [16].

In conclusion, we here present a first detailed analysis of the global Ebola research landscape. The collected data indicated that the efforts in scientific research have been constantly increasing since the time of discovery in 1976. The USA was identified as being the leading country and a total of more than 3000 publications.

However, the danger of the virus, the change in pattern of distribution and the neglect to put more emphasis on the development vaccines before the outbreak of 2013–2015 clearly point to the need that 1) research in the field of hemorrhagic fevers needs to be strengthened, 2) vaccine development should also be enforced for other neglected tropical diseases in order to prevent similar catastrophes in the future, and 3) research endeavors should be focused on the area of public health since we could identify a neglect in Ebola related public health research efforts.

## References

[1] Baize S, Pannetier D, Oestereich L, Rieger T, Koivogui L, et al (2014) Emergence of Zaire Ebola virus disease in Guinea. N Engl J Med 371: 1418–1425. 10.1056/NEJMoa1404505 24738640

[2] Wolf T, Kann G, Becker S, Stephan C, Brodt HR, et al (2014) Severe Ebola virus disease with vascular leakage and multiorgan failure: treatment of a patient in intensive care. Lancet. 10.1016/S0140-6736(14)62384-9 25534190

[3] Leroy EM, Kumulungui B, Pourrut X, Rouquet P, Hassanin A, et al (2005) Fruit bats as reservoirs of Ebola virus. Nature 438: 575–576. 16319873 10.1038/438575a

[4] Bausch DG (2014) One Step Closer to an Ebola Virus Vaccine. N Engl J Med. 10.1056/NEJMe1414305 25426836

[5] Dawson AJ (2015) Ebola: what it tells us about medical ethics. J Med Ethics 41: 107–110. 10.1136/medethics-2014-102304 25516949

[6] Gastner MT, Newman ME (2004) From The Cover: Diffusion-based method for producing density-equalizing maps. Proc Natl Acad Sci U S A 101: 7499–7504. 15136719 10.1073/pnas.0400280101 PMC419634

[7] Groneberg-Kloft B, Quarcoo D, Scutaru C (2009) Quality and quantity indices in science: use of visualization tools. EMBO Rep 10: 800–803. 10.1038/embor.2009.162 PMC2726685 19648952

[8] Fricke R, Uibel S, Klingelhoefer D, Groneberg DA (2013) Influenza: a scientometric and density-equalizing analysis. BMC Infect Dis 13: 454 10.1186/1471-2334-13-454 24079616 PMC3851602

[9] Hirsch JE (2005) An index to quantify an individual's scientific research output. Proc Natl Acad Sci U S A 102: 16569–16572. 16275915 10.1073/pnas.0507655102 PMC1283832

[10] Fergusson W (1817) An inquiry into the Origin and Nature of the Yellow Fever, as it has lately appeared in the West Indies, with Official Documents relating to this subject. Med Chir Trans 8: 108–172. 20895312 10.1177/095952871700800105 PMC2129003

[11] Smart WR (1877) On Dengue or Dandy Fever. Br Med J 1: 382–383. 20748489 10.1136/bmj.1.848.382 PMC2220391

[12] (1978) Ebola haemorrhagic fever in Sudan, 1976. Report of a WHO/International Study Team. Bull World Health Organ 56: 247–270. 307455 PMC2395561

[13] Centers for Disease C, Prevention (1995) Outbreak of Ebola viral hemorrhagic fever—Zaire, 1995. MMWR Morb Mortal Wkly Rep 44: 381–382. 7739512

[14] Groneberg-Kloft B, Scutaru C, Kreiter C, Kolzow S, Fischer A, et al (2008) Institutional operating figures in basic and applied sciences: scientometric analysis of quantitative output benchmarking. Health Res Policy Syst 6: 6 10.1186/1478-4505-6-6 18554379 PMC2459159

[15] Feldmann H (2006) Marburg hemorrhagic fever—the forgotten cousin strikes. N Engl J Med 355: 866–869. 16943398 10.1056/NEJMp068160

[16] Bundschuh M, Groneberg DA, Klingelhoefer D, Gerber A (2013) Yellow fever disease: density equalizing mapping and gender analysis of international research output. Parasit Vectors 6: 331 10.1186/1756-3305-6-331 24245856 PMC3843536

[17] Al-Mutawakel K, Scutaru C, Shami A, Sakr M, Groneberg DA, et al (2010) Scientometric analysis of the world-wide research efforts concerning Leishmaniasis. Parasit Vectors 3: 14 20202187 10.1186/1756-3305-3-14 PMC2845575

[18] Garg KC, Kumar S, Madhavi Y, Bahl M (2009) Bibliometrics of global malaria vaccine research. Health Info Libr J 26: 22–31. 10.1111/j.1471-1842.2008.00779.x 19245640

[19] Adams J (2013) The fourth age of research. Nature 497: 557–560. 10.1038/497557a 23719446

[20] Gunther S, Feldmann H, Geisbert TW, Hensley LE, Rollin PE, et al (2011) Management of accidental exposure to Ebola virus in the biosafety level 4 laboratory, Hamburg, Germany. J Infect Dis 204 Suppl 3: S785–790. 10.1093/infdis/jir298 21987751

